# The Emergence of Inner Speech and Its Measurement in Atypically Developing Children

**DOI:** 10.3389/fpsyg.2020.00279

**Published:** 2020-03-17

**Authors:** Constance Th. W. M. Vissers, Ekaterina Tomas, James Law

**Affiliations:** ^1^Royal Dutch Kentalis, Sint-Michielsgestel, Netherlands; ^2^Behavioural Science Institute, Radboud University, Nijmegen, Netherlands; ^3^National Research University – Higher School of Economics, Moscow, Russia; ^4^School of Education, Communication and Language Sciences, Newcastle University, Newcastle upon Tyne, United Kingdom

**Keywords:** inner speech, covert speech, language, children, developmental language disorder, autism, hearing loss

## Abstract

Inner speech (IS), or the act of silently talking to yourself, occurs in humans regardless of their cultural and linguistic background, suggesting its key role in human cognition. The absence of overt articulation leads to methodological challenges to studying IS and its effects on cognitive processing. Investigating IS in children is particularly problematic due to cognitive demands of the behavioral tasks and age restrictions for collecting neurophysiological data [e.g., functional magnetic resonance imaging (fMRI) or electromyography (EMG)]; thus, the developmental aspects of IS remain poorly understood despite the long history of adult research. Studying developmental aspects of IS could shed light on the variability in types and amount of IS in adults. In addition, problems in mastering IS might account for neuropsychological deficits observed in children with neurodevelopmental conditions. For example, deviance in IS development might influence these children’s general cognitive processing, including social cognition, executive functioning, and related social–emotional functioning. The aim of the present paper is to look at IS from a developmental perspective, exploring its theory and identifying experimental paradigms appropriate for preschool and early school-aged children in Anglophone and Russian literature. We choose these two languages because the original work carried out by Vygotsky on IS was published in Russian, and Russian scientists have continued to publish on this topic since his death. Since the 1960s, much of the experimental work in this area has been published in Anglophone journals. We discuss different measurements of IS phenomena, their informativeness about subtypes of IS, and their potential for studying atypical language development. Implications for assessing and stimulating IS in clinical populations are discussed.

## Inner Speech From a Developmental Perspective

“*There is no doubt that specifically human cognition is completely intertwined with speech.*”

Galperin (1957)

**FIGURE 1:**
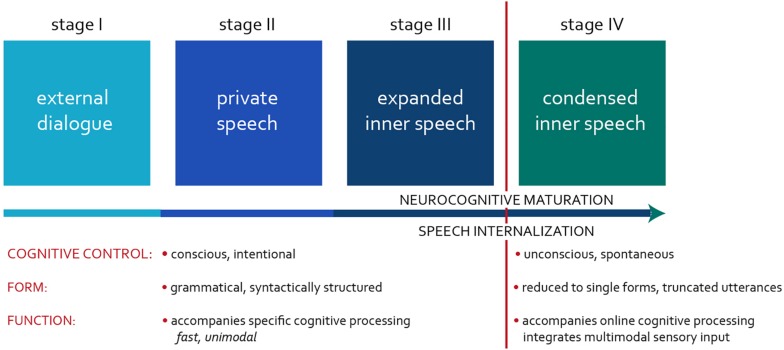
Schematic representation of stages of IS development.

“Inner speech” (IS) was a term originally coined by the Russian psychologist Lev Vygotsky to capture the process by which the private speech (PS) of young children, talking to themselves out loud during play, starts accompanying their activity in a variety of cognitive tasks (Vygotsky, 1934). IS results from gradual internalization of overt speech in children, comprising three stages in Vygotsky’s original model (1934, 1986). In our paradigm, we split the final stage into two (Figure 1).

Stage I occurs during early language acquisition when children master the fundamentals of an *external dialogue* (ED). It focuses on *connecting with others* – on communication and regulation of one another’s behavior.

Around the age of 3–4 years, as children’s linguistic experience increases, they enter Stage II and start talking to themselves (Winsler et al., 2000). This phenomenon is known as PS, when the child attempts to imitate an adult talking to them, thereby regulating their behavior. At this stage, the main function of PS is *self-regulation* or *self-guidance* (Berk and Garvin, 1984): children “whisper” to themselves planning their next step or commenting on their current activity. A distinguishing feature of PS compared to ED is the absence of an interlocutor, which allows simplifying compositional and syntactic conventions required in a dialogue with an interlocutor. However, the linguistic aspects of PS remain unexplored and require further study. ED and PS also share similarities: they represent *overt* speech and involve conscious control, focusing on the current, planned, or sometimes recalled event. Despite variability in the amount of PS observed in children, it is universally used across languages (Vygotsky, 1934; Berk and Garvin, 1984; Winsler et al., 2000, 2003; Al-Namlah et al., 2006).

The flexibility in using speech *covertly* develops after the age of 6–7 years (Vygotsky, 1934), when children fully internalize their thoughts during various cognitive tasks, such as silent remembering, reading, and writing. In Vygotsky’s model, this occurs during Stage III, suggesting the *full mastery of IS*. Following this paradigm, the studies on IS have explored a wide variety of phenomena involving covert self-talk, ranging from silent reading and mental arithmetic (i.e., so-called “speech minus sound”; Müller, 1864) to unconscious “thinking in a language.” Alderson-Day and Fernyhough (2015) have recently introduced the terms “expanded” and “condensed” IS to differentiate between these typologically distinct phenomena. We adopt their approach throughout the paper: Stage III represents the development of expanded, and Stage IV, condensed IS.

Expanded IS often occurs during linguistic tasks, such as silent reading and writing, or mental rehearsal of a dialogue. This type of IS shares similarities with PS, as both activities are task-driven and *conscious*. The latter makes it possible to easily recall the content of the recent PS/expanded IS event. Since PS and expanded IS are task-driven, they focus on current or planned activity, representing *top–down processes*. Finally, both PS and expanded IS involve linguistically well-formed, grammatical utterances. Adults often use expanded IS and PS interchangeably, switching from covert to overt speech, depending on the situational context. Interestingly, based on experience sampling questionnaires, adults are estimated to engage in expanded IS approximately 20% of the time (e.g., Heavey and Hurlburt, 2008), suggesting that this form of IS coexists with condensed IS during cognitive processing.

Condensed IS represents the final stage of speech internalization. It is a *fluid*, *spontaneous*, and *unconscious* process, during which an utterance is often *reduced to a single grammatical form* (Vygotsky, 1934; Galperin, 1957; Sokolov, 1967) associated with the current experience. This type of covert speech intertwines with human thinking, occurring *spontaneously* and *unconsciously*. It resembles a *bottom–up* perception of sensory input, most of which is processed automatically through implicit/unconscious neural mechanisms. The attentional account of multisensory processing claims that integrating information coming from different modalities is dependent on both top–down and bottom–up processes and that our mental representations of the surrounding environment are shaped by internal cognitive processes and the sensory input (Talsma, 2015). The dual nature of IS – its interplay between top–down and bottom–up processing – suggests its possible role in integrating multisensory information into internally consistent mental representations. Recent neuroscientific evidence supports this: the neuroanatomic substrates engaged in multisensory processing, such as parts of the parietal [angular gyrus – Brodmann area (BA) 39] and temporal cortex (BA 20, BA 37, BA 38), are also involved in language functioning (Seghier, 2013; Ardila et al., 2016).

Children not only internalize but also contract or abbreviate their IS over time. The more familiar and automatic the cognitive process/task becomes, the more abbreviated (and thus more condensed) is the accompanying IS (Galperin, 1957). The complexity of cognitive tasks also contributes to the IS involvement in adults and children (Sokolov, 1967; Fatzer and Roebers, 2012). In more cognitively demanding tasks, articulatory suppression has a detrimental effect on performance because it debilitates IS. This evidence supports the integrative role of IS in multisensory processing. It also explains why children, who have less cognitive resources and control than adults, prefer less abbreviated PS and expanded IS, particularly during novel cognitively demanding tasks.

## Is Effects on Cognitive Functioning and Cognitive Development

Alderson-Day and Fernyhough (2015) have summarized findings on the role of IS during cognitive processing, highlighting its effects on executive functions, including short-term memory (Williams et al., 2012) planning (Al-Namlah et al., 2006; Lidstone et al., 2010), control of behavior (Cragg and Nation, 2010; see also DeGraaf and Schlinger, 2012), inhibition, and cognitive flexibility (Fatzer and Roebers, 2012). IS also supports Theory of Mind (Fernyhough and Meins, 2009), communicative and social interactions, self-awareness, self-monitoring, motivation, and creativity (Brinthaupt et al., 2009; Barkley, 2012; Alderson-Day and Fernyhough, 2015).

Additional evidence on the interactive relationship between cognitive processing and IS comes from clinical populations. For example, adults with aphasia (Feinberg et al., 1986; Geva, 2010; Farrar et al., 2009; Geva et al., 2011b; Langland-Hassan et al., 2015) do not rely on IS during cognitive tasks to the same extent as their unimpaired peers. However, the interplay between IS and verbal skills in adult clinical populations is unclear: some patients with aphasia demonstrate better preserved IS abilities compared to their overt speech, and others show the opposite pattern (Farrar et al., 2009; Geva et al., 2011a). The multifaceted nature of covert speech suggests that the dissociation between IS and overt speech in these individuals arises from different types of deficits. It might be the case that a patient with aphasia is suffering from only condensed IS deficits or that both expanded and condensed IS are impaired. The distinction between different subtypes of IS phenomena may therefore help account for heterogeneity in neurocognitive profiles and behavioral phenomenology observed in typical and clinical populations. It is also possible, of course, that measurement issues, which are key to the assessment of IS may be especially salient when it comes to atypical populations.

Interactions between language development, cognitive development, and behavioral problems have been reported for children with atypical language profiles – related to developmental language disorder (DLD), hearing loss, and autism (e.g., Jamieson, 1995; Wallace et al., 2009; Lidstone et al., 2012; Vissers et al., 2018). Communication with these children can be challenging, leading to insufficient input and language practice and subsequent social isolation. This contributes to delays in Theory of Mind (ToM) Development, executive deficits, and related social–emotional disorders (Vissers et al., 2015; Vissers et al., 2016). Consistent with this assumption are studies showing that deaf children of deaf parents who communicate in sign language from birth and hence have less difficulty constructing adequate social dialogues appear to follow undisrupted development of sign language internalization and self-regulation (Vissers et al., 2018). For instance, Hall et al. (2017), working with deaf parents of deaf children, who had exposed their child to a natural sign language from birth, asked them to complete the parent-report Behavior Rating Inventory of Executive Function (BRIEF) about their children and found that the children, on average, received age-appropriate scores on all of the executive function domains assessed by the BRIEF (inhibitory control, flexibility, emotional control, initiate, working memory, plan/organize, organization of materials, and monitor). Similarly, deaf and hard-of-hearing children raised by deaf compared to hearing mothers demonstrate more mature PS (i.e., self-directed covert signing) and its more frequent use (Jamieson, 1995). Based on questionnaire data, more private signing and increased positive/motivational PS is also observed in congenitally deaf adults (Zimmermann and Brugger, 2013), raising questions about typological differences in IS across spoken and sign languages.

Delay or deviance in IS development has been reported for 7- to 10-year-old children with DLD (Lidstone et al., 2012). Although at this age, children with DLD have shown normal effects of articulatory suppression on a Tower of London task, overall, their PS was less internalized compared to controls, indicating a delay in their IS development reflecting that in their external expressive and receptive language. These deficits possibly account for the poorer performance of the DLD group on the Tower of London task despite similar non-verbal IQ scores across groups.

Studying speech internalization in children with atypical language development (i.e., the status of their PS, expanded IS, and condensed IS) could contribute to tailored assessment and intervention. For example, a recent intervention study has demonstrated that self-regulatory speech training, which is analogous to PS stimulation, can improve planning and problem-solving performance in children with DLD (Abdul Aziz et al., 2016), suggesting environmental origins of IS and direct implications for future clinical practice.

To summarize, it appears that IS optimizes cognitive performance in adults and accounts for cognitive deficits in children with DLD, hearing loss, and autism, although it is difficult to anticipate the detail of qualitatively different manifestations of IS across clinical populations. Impaired overt speech (“communication with others”) leads to disruptions in PS and IS throughout the speech internalization process, but more evidence is needed to explore the fine-grained differences in the IS profiles across clinical populations.

## Direct and Indirect Measurements of Is

Researchers have long investigated IS directly and indirectly using behavioral (Emerson and Miyake, 2003; Miyake et al., 2004; Holland and Low, 2010; Lidstone et al., 2010; Fatzer and Roebers, 2012; also see for review Alderson-Day and Fernyhough, 2015) and cognitive experiments (Morin and Michaud, 2007; Geva et al., 2011b; Tian and Poeppel, 2012).

Behavioral experiments rely on encouraging IS in participants and explore the quality and quantity of IS across individuals. They involve verbal reporting on recent IS experiences as they spontaneously occur in daily life. These paradigms encompass classical questionnaires or experience sampling (McCarthy-Jones and Fernyhough, 2011; Morin et al., 2011; Alderson-Day and Fernyhough, 2015; Hurlburt and Heavey, 2015). While questionnaires force participants to endorse pre-existing IS content, sampling methods require reporting specific aspects of inner experiences at random – as a reaction to an external signal, e.g., a beep. Importantly, the studies demonstrate a lot of variability in the amount and the quality of IS reported by participants (Ren et al., 2016). However, this may be due to the participants’ reflection abilities rather than to individual variability in the amount of covert speech. Questionnaires and experience sampling methods involve direct reports on IS experiences, and both methods likely tap into expanded IS because they require reporting consciously memorized events.

Alternative indirect behavioral methods come from the cognitive literature and include protocol analysis and the “silent dog” paradigm (Hayes et al., 1998; Alvero and Austin, 2006; Arntzen et al., 2009). Both methods involve training the participants to verbalize their thoughts when performing a non-verbal task and explore whether the resulting self-talk helps in controlling their behavior. The advantage of these paradigms is that they control for variability in the amount of reported IS experiences compared to questionnaires and experience sampling. However, the ecological validity of this approach for exploring condensed IS and for differentiating between PS and expanded IS remains unclear: the participants are aware that others observe and record their self-talk, and thus, their verbalizations are likely to be fully grammatical and intelligible utterances compared to the truncated sentences typical for IS.

Cognitive methods include dual-task paradigms (Coltheart and Langdon, 1998; Emerson and Miyake, 2003; Miyake et al., 2004; Wallace et al., 2009; Holland and Low, 2010; Lidstone et al., 2010; Fatzer and Roebers, 2012), involving suppression of covert speech when the participant is performing another cognitive task (such as logical reasoning) or blocking covert speech by presenting items at a fast rate. These paradigms assume that blocking articulation impedes linguistic processing in general, including IS. Negative effects of articulatory suppression on task performance suggest that participants cannot rely on IS to optimize their cognitive processing. One limitation of this paradigm is its indirect nature: participants perform two unfamiliar cognitive tasks, which increases the cognitive load. Thus, any increase in reaction time, or decrease in accuracy, may be due to cognitive difficulty performing a dual task. This approach also cannot separate the effects of expanded vs. condensed IS on task performance.

A second cognitive method is a dual-task paradigm involving a linguistic task, such as silent rhyming, which requires subvocalization (Levine et al., 1982; Feinberg et al., 1986; Geva et al., 2011a, b; Langland-Hassan et al., 2015). Since this type of task involves focused activity, it is likely to measure predominantly expanded IS and does not tap into the spontaneous and fluid unconscious phenomenon of condensed IS. Similar to questionnaires, dual-task paradigms with linguistic tasks cannot explore the role of IS in cognitive processing directly and are particularly vulnerable to linguistic constraints especially when the child’s language development is immature or disordered.

The neural substrates governing IS can be investigated with neurophysiological measurements. The neurophysiological signatures of overt vs. covert naming have been explored in positron emission tomography (PET) studies. For example, the participants saw written words and pictures of objects in the scanner and were instructed to read the words and name the objects covertly and overtly (e.g., Bookheimer et al., 1995). Overt naming of objects produced very similar patterns of neural activation to covert naming of objects, except for regions associated with motor activity. Generally, studies comparing overt and covert speech have found somewhat mixed results, suggesting that overt speech cannot be conceptualized as covert speech plus motor and auditory cortex activation (*inter alia* Huang et al., 2001; Shuster and Lemieux, 2005). Until now, overt and covert speech have not been compared in the same study or under the same experimental conditions, limiting the generalizability of these findings.

Recent fMRI studies investigated IS in healthy adult participants, requiring them to silently complete sentences (Friedman et al., 1998; Sherrgill et al., 2001). Similarly, Bullmore et al. (2000) presented single words on a screen, asking the participants to covertly articulate their semantic judgment on the animacy of the stimulus, i.e., whether the word indicated a living or non-living object. Activation was found for the ventral extrastriate and prefrontal cortices governing word recognition and semantic processing, and for the prefrontal cortex and Broca’s area related to (subvocal) planning and articulation. Similarly, activation in the inferior parietal lobule, precuneus, and temporal gyrus presumably represents monitoring of Broca’s area output. This method, therefore, has the potential for disentangling the neural correlates of expanded and condensed IS (see Jones, 2009).

An alternative neurophysiological method for studying IS is electromyography (EMG), which can be used for measuring activation/tenseness of articulatory organs (Sokolov, 1967). In a series of experiments using a dual-task paradigm with adults and children, Sokolov has demonstrated that tenseness of articulatory organs increases when performing cognitively demanding and unfamiliar tasks, supporting the idea that IS optimizes cognitive processing. More recent EMG studies confirm that IS is accompanied by activity in the orofacial musculature (Loevenbruck et al., 2018). For example, Livesay et al. (1996) reported an increase in EMG activity during silent recitation compared to rest but no increase during a non-linguistic visualization task. Nalborczyk et al. (2017) reported an increase in labial EMG activity during rumination (having negative thoughts during IS) compared with relaxation. To summarize, the EMG paradigm combining behavioral and neurophysiological methods is another alternative for exploring expanded and condensed IS, using an ecologically valid experimental design.

## Conclusion

Inner speech serves as a valuable concept that has withstood the test of time since it was first articulated by Vygotsky and his colleagues. The covert nature of IS makes it challenging to study, and particularly to disentangle typologically distinct phenomena, such as expanded and condensed IS. Behavioral, cognitive, and neurophysiological paradigms have made progress exploring covert speech in adults, but few of them could be used with children, including preschoolers and those from atypical populations. This suggests that we need to use a modified combination of the existing paradigms in order to study IS from developmental perspective. For example, instead of fMRI, one could use more child-friendly electroencephalography (EEG) in combination with EMG to measure neurophysiological activity during cognitive and linguistic tasks.

The area that has the most potential for future research is the study of IS in children with neurodevelopmental disorders, because in such conditions, children often experience deficits in expressive and receptive language skills in combination with self-regulation and Theory of Mind problems (both clearly associated with IS). At present, it is impossible to formulate specific hypotheses about the likely manifestations of IS deficits across clinical conditions, such as DLD, autism, and hearing loss. For example, it is unknown to what extent children with different developmental deficits rely on expanded and condensed IS during cognitive processing, and we know little about the role their specific speech and language deficits play in their IS profiles. For example, is IS level a function of individual variability or is it driven by expressive or receptive language levels or other aspects of cognition, and how sensitive are these differences to the features of specific disorders, for example, Theory of Mind deficits in autism or phonological deficits in DLD? Studying the development and functions of overt speech in these children is important from both theoretical and clinical perspectives. For example, stimulating IS development during intervention might enhance the cognitive and linguistic efficacy of the program. These findings are also important for fundamental research. The comparison of IS in typical and atypical development has the potential to inform our understanding of this uniquely human phenomenon.

## Author Contributions

All authors listed have made a substantial, direct and intellectual contribution to the work, and approved it for publication.

## Conflict of Interest

The authors declare that the research was conducted in the absence of any commercial or financial relationships that could be construed as a potential conflict of interest.
